# Methods to safely implement hypothermia in the intensive care unit: a how-to guide

**DOI:** 10.5935/0103-507X.20160007

**Published:** 2016

**Authors:** Christian Storm, Natalie M. Otto

**Affiliations:** 1 Charité Universitätsmedizin Berlin - Berlin, Germany.

## TARGET TEMPERATURE MANAGEMENT IN 2016

Target temperature management (TTM) is well-known to reduce secondary cell damage after cardiac arrest in patients with presumed cerebral hypoxia. The treatment of this reperfusion syndrome, especially in terms of temperature management, is not fully understood. Several clinical randomized controlled trials and other studies have shown TTM's effectiveness in improving neurological outcomes.^(1)^ Therefore, TTM has been recommended in the updated guidelines of the European Resuscitation Council since October 2015.^(2)^ Briefly, TTM is indicated for almost all survivors after cardiac arrest with different levels of evidence supporting this claim. The initial rhythm (shockable or non-shockable) and the location of the arrest (in-/out-of-hospital cardiac arrest) do not exclude treatment if the patient remains comatose after the return of spontaneous circulation. Due to new published data, the recommended target temperature is between 32°C and 36°C for 24 hours; the optimal target temperature and duration of cooling is still unknown.^(3)^ In addition, modern TTM is only a part of the care for the post-cardiac arrest phase, including early percutaneous coronary intervention, optimization of blood glucose levels and ventilator setup to achieve normoxia and normocapnia. Several surveys in Europe revealed an increasing acceptance of TTM after cardiac arrest, but there is still an intermediate rate of using professional computer feedback guided temperature management and the standard operating protocol (SOP).^(4-6)^ The reasons behind the reluctance are numerous. When comparing the benefit of using TTM with neurological outcomes and recovery, some of these reasons will no longer acceptable in the future.

## IMPLEMENTATION OF TARGET TEMPERATURE MANAGEMENT IN YOUR INTENSIVE CARE UNIT

### The team

The slogan "KISS" is key to your success and stands for: keep it sweet and simple. As easy as it sounds, the truth is that your success is about your team and the teamwork needed when implementing new treatments in the intensive care unit (ICU). It is important to convince your team of the new method, explain current data and guidelines, and define stakeholders (nurse/physician) and responsible team members for providing training and answering questions of new staff. It is also important to choose the most appropriate cooling device (computer feedback) for your ICU and offer repetitive training on the device. Your stakeholder is very important as he or she is part of your team already, and the team will easily accept training and advice from their own members that are on the same level.

### Standard operating protocol

Providing a written SOP is important and should include the criteria for patients who are receiving TTM and exclusion criteria for exceptions; it should also include the indicated target temperature and duration of TTM. A major part of the SOP should be a list of side effects and problems that can occur during treatment and how to avoid, detect and or treat them successfully. During the implementation phase, the SOP and trouble-shooting list should be discussed with your team and adjusted in accordance with their wishes. Typical side effects and their appropriate treatments should be mentioned, such as bradycardia, hypokalemia or shivering. For example, the SOP should advise that counter-warming (gloves and socks) and deep sedation will effectively prevent shivering in most patients if started prior to the occurrence of shivering. If shivering has already been observed, a list of detailed steps for treatment solutions, such as deepening sedation, intravenous magnesium application, and counter-warming, should be provided; if shivering is persistent, muscle paralysis is an important point to mention. Modify your SOP with your team's input, and the SOP will not only enhance the quality of care but also give your team a safer attitude towards implementation of TTM. Engaging your staff at these important steps of implementation will significantly increase the level of acceptance and usage. Additionally, if your team does well, you should tell them. Feedback is another very important key for success and motivation.

### The first patients

If possible, invite discharged survivors after cardiac arrest to visit your team as a form of positive feedback. Your first results will also convince the still skeptic team members. It is important that all staff members adopt the new method to guarantee that all patients will receive the best medical care according to the local SOP after a cardiac arrest.

### Neurological prognostication

**Figure 1:**
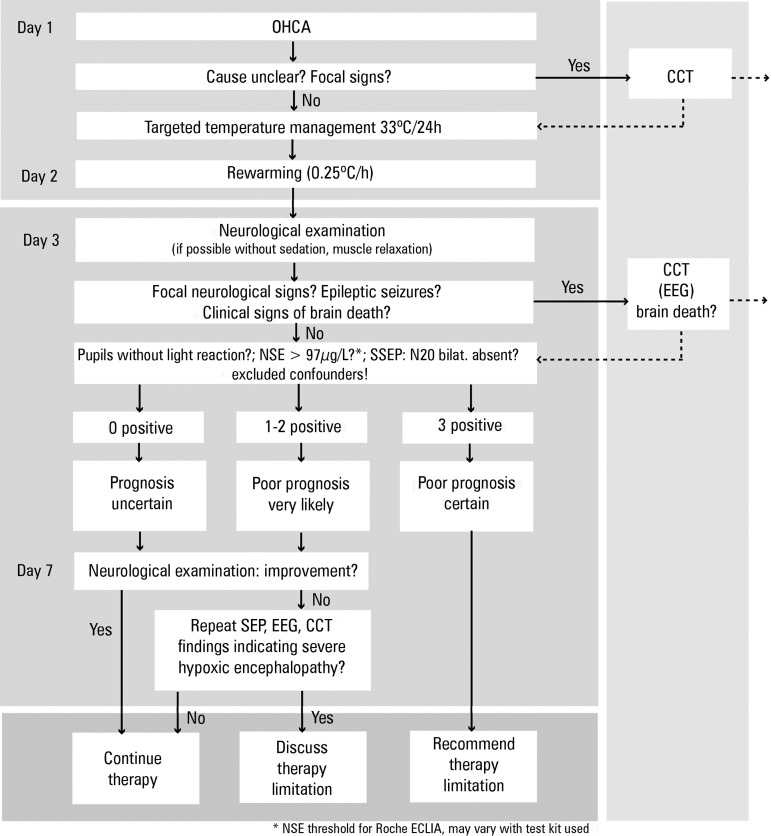
The local algorithm for neurological prognostication designed and used at the Charité Universitätsmedizin Berlin hospital. The pathway includes current recommendations of several intensive care and neurological societies. OHCA - out-of-hospital cardiac arrest; EEG - electroencephalography; SEP - somatosensory evoked potentials; Cct - cerebral computer tomography; NSE - neuron specific enolase.

Prognostication has changed remarkably over the last decade. As recommended by current guidelines, a multimodal process of prognostication, including biomarkers such as neuron specific enolase, clinical examination, somatosensory evoked potentials, electroencephalography and computer tomography are useful and important.^(7)^ In addition to this approach, it is clearly recommended not to start prognostication too early to exclude confounders, such as residual sedation. Prognostication should take place between days 3-7. In the case of different or conflicting results, ongoing observation of the patient and re-evaluation is recommended. However, your local SOP should also highlight the important pathways for prognostication and which steps are used for your patients under which conditions (Figure 1). For successful implementation of a prognostication pathway, a close collaboration with a Neurology department is recommended, if possible.

## CONCLUSION

There is no doubt regarding the need for TTM after cardiac arrest to improve neurological outcomes. It is important to define key members of the team and provide a written SOP that includes trouble-shooting, including your team in all the steps of implementation and modifying the SOP according to the team's input. Remember to give feedback after your first patients to motivate your team. Successful implementation of TTM requires teamwork and to "KISS".
